# Structured surveillance in Lynch syndrome: effectiveness, limitations, and unmet needs

**DOI:** 10.1016/j.esmogo.2026.100399

**Published:** 2026-08-10

**Authors:** A. Dardenne, R. Cohen, C. Evrevin, C. Duros, N. Basset, P. Cervera, J.H. Lefevre, T. Germain, C. Lepillier, N. Chabbert-Buffet, T. André, X. Dray, Y. Parc

**Affiliations:** 1 Department of Medical Genetics, Sorbonne University, Pitié-Salpêtrière Hospital, Assistance Publique-Hôpitaux de Paris, Paris, France; 2 Microsatellite Instability and Cancers Research Laboratory, INSERM and Sorbonne University, Centre de Recherche Saint-Antoine, Paris, France; 3 Department of Digestive Surgery, Sorbonne University, Saint-Antoine Hospital, Assistance Publique-Hôpitaux de Paris, Paris, France; 4 Department of Medical Oncology, Sorbonne University, Saint-Antoine Hospital, Assistance Publique-Hôpitaux de Paris, Paris, France; 5 Department of Gynecology, Sorbonne University, Tenon Hospital, Assistance Publique-Hôpitaux de Paris, Paris, France; 6 Oncogenetics Unit, Saint-Louis Hospital, Assistance Publique-Hôpitaux de Paris, Paris, France; 7 Department of Pathology, Sorbonne University, Pitié-Salpêtrière Hospital, Assistance Publique-Hôpitaux de Paris, Paris, France; 8 Gut, Liver & Microbiome Research (GLIMMER) FHU, Paris, France; 9 Department of Urology, Sorbonne University, Tenon Hospital, Assistance Publique-Hôpitaux de Paris, Paris, France; 10 Department of Gynecology, Sorbonne University, Pitié-Salpêtrière Hospital, Assistance Publique-Hôpitaux de Paris, Paris, France; 11 Sorbonne University, Center for Digestive Endoscopy, Saint-Antoine Hospital, Assistance Publique-Hôpitaux de Paris, Paris, France

**Keywords:** colorectal cancer, gastrointestinal, Lynch syndrome, mismatch repair genes, surgery, surveillance

## Abstract

**Background:**

Lynch syndrome confers a high lifetime risk of multiorgan cancers. Although colorectal surveillance is well established, prospective data on cancer incidence and adherence to structured multiorgan follow-up and surveillance recommendations remain limited.

**Materials and methods:**

We retrospectively analyzed prospectively collected data from 517 individuals carrying a pathogenic or likely pathogenic variant in a mismatch repair gene at a single specialized French center. All patients underwent structured surveillance, including regular colonoscopy and organ-specific follow-up according to national guidelines. Cancer incidence rates, tumor distribution, and modes of detection (asymptomatic versus symptomatic) were assessed over the follow-up.

**Results:**

Over 4827 person-years, 201 cancers were diagnosed (annual cancer incidence 4.16%, 95% confidence interval 3.59-4.74). Colorectal cancer was most frequent (41.8%), followed by urinary tract (11.9%) and skin cancers. Among guideline-surveilled organs (*n =* 132), most were detected asymptomatically, particularly lower gastrointestinal (GI) (84.5%) and urinary tract (85%) cancers, whereas >50% of upper GI cancers were diagnosed symptomatically. Gynecologic cancers were rare, likely reflecting prophylactic surgery. Notably, 69 cancers (34%) arose outside guideline-recommended surveillance organs, primarily skin, breast, prostate, and pancreas. Cancer-specific mortality was lower among the cancers detected under surveillance, although this difference did not reach statistical significance.

**Conclusions:**

Despite structured, multiorgan surveillance, patients with Lynch syndrome exhibit high cancer incidence and broad tumor distribution. Although current protocols enable early detection for several organs, a substantial proportion of cancers occur outside existing recommendations. These findings support follow-up in dedicated coordination centers and may inform the selective expansion of surveillance strategies to additional high-risk organs.

## Highlights

• Annual cancer incidence was 4.16% across 4827 person-years in 517 MMR variant carriers.
• Surveillance enabled high asymptomatic detection of colorectal and urinary tract cancers.
• Upper GI and gynecologic cancers were often diagnosed symptomatically despite surveillance.
• More than one-third of cancers occurred outside guideline-surveilled organs.
• Findings support broader, multidisciplinary surveillance strategies in Lynch syndrome.

## Introduction

The follow-up of individuals with a hereditary predisposition to cancer due to pathogenic variants in mismatch repair (MMR) genes represents a major clinical challenge. With an estimated prevalence ranging from 1 in 550 to 1 in 350 in the general population,^1^^,^^2^ carriers of these variants have a markedly increased risk of developing cancer, often with early onset, and in some cases, multiple or recurrent tumors.

The aims of surveillance are twofold: to prevent cancer development and to facilitate early detection through personalized, multidisciplinary, and regular follow-up. In response to these challenges, specialized care structures dedicated to the management of high-risk individuals have been established in France, integrating national clinical guidelines and ensuring close coordination across medical specialties.

Despite the increasing identification of individuals diagnosed with Lynch syndrome, prospective follow-up data in this high-risk population remain scarce in France.^3^ Although colorectal surveillance is well established, international consensus is limited regarding optimal surveillance strategies for other organ systems, largely due to a lack of robust evidence.

This study reports a retrospective analysis of prospectively collected data from a single prevention-oriented center. All included individuals were confirmed carriers of a pathogenic or likely pathogenic (P/LP) variant in one of the MMR genes associated with Lynch syndrome and were followed according to a structured surveillance protocol.

The primary objective of this study was to estimate the incidence of cancers diagnosed during prospective follow-up in this genetically defined population. The secondary objective was to evaluate the concordance between the observed cancers and current organ-specific surveillance recommendations as defined in regional French guidelines.

## Materials and methods

### Study design and population

This was a retrospective analysis of a prospectively followed, single-center cohort conducted at Saint-Antoine Hospital, Paris, France. The study cohort consisted of 517 individuals carrying a P/LP variant in one of the *MMR* genes. Eligible individuals were aged >25 years and had undergone prospective follow-up, including at least one planned colonoscopy. Inclusion criteria were consistent with those of the Prospective Lynch Syndrome Database (PLSD).^4^ The age at inclusion was defined as the age at which the first prospectively planned and completed colonoscopy was carried out. In earlier years, inclusion was based on family history rather than confirmed genetic testing. In some cases, the pathogenic variant was therefore identified retrospectively; however, these patients were enrolled in structured follow-up based on clinical criteria. Follow-up time was calculated from the date of inclusion. Cancers diagnosed before the initiation of prospective follow-up were excluded from the present analyses.

### Data collection and ethical considerations

Prédisposition Digestive Ile-de-France (PRED-IdF) network is a French regional network in the greater Paris area funded by the French National Cancer Institute in 2009 to support patients carrying a genetic predisposition to gastrointestinal (GI) cancers in their ongoing follow-up. Patients were identified through the prospective PRED-IdF local database, which included all individuals with a genetic predisposition to GI cancers between March 2010 and July 2024. The study was registered in the institutional database under the reference code APHP251211, in accordance with French data protection regulations (MR004 framework, non-Jardé study).

Clinical data were retrieved from electronic medical records, specialist consultation notes, and radiological reports. All patients provided written informed consent for both genetic testing and enrollment in the long-term surveillance program.

Germline variants were interpreted following the latest American College of Medical Genetics and Genomics guidelines. Only germline variants classified as P or LP were included.

Patients with so-called ‘Lynch-like syndrome’, characterized by a suggestive family history or early-onset tumors exhibiting MMR deficiency but lacking an identifiable germline pathogenic variant or evidence of MMR gene promoter hypermethylation, were excluded. Variants of uncertain significance were also excluded. Cancer types were classified according to the *International Classification of Diseases, 10th Revision* (2019 version).

The structured surveillance protocol included upper GI endoscopy every 4 years, dye-spray colonoscopy every 2 years, and urinary tract monitoring, including urinalysis, urinary cytology, and pelvic ultrasound for *MSH2* mutation carriers and individuals with a family history of urinary tract cancers. In cases of microscopic hematuria, a repeat urine sample was obtained after 1 month to confirm persistence. For female patients, annual clinical examination, pelvic ultrasound, and endometrial biopsy were carried out.

### Statistical analysis

Descriptive statistics were used to summarize patient characteristics, genetic variants, and cancer types. Categorical variables are reported as counts and percentages and continuous variables as medians with interquartile ranges (IQRs). Annual cancer incidence rates were calculated as the number of cancers diagnosed during follow-up divided by the total number of person-years at risk. Follow-up duration is expressed in years and was computed for each patient from inclusion to last follow-up. Confidence intervals (CIs) for incidence rates were estimated assuming a Poisson distribution of events. Cancer-free survival from inclusion was estimated using the Kaplan–Meier method, stratified by MMR gene.

### Outcome measures

The primary outcome was the annual cancer incidence rate, calculated as (total number of cancers / total person-years of follow-up) × 100 during prospective follow-up. The secondary outcome was the proportion of cancers detected asymptomatically versus symptomatically. Cancers were classified as symptomatic if they were diagnosed according to clinical symptoms that prompted diagnostic investigations outside the scheduled surveillance protocol.

## Results

### Baseline characteristics and follow-up data

**Figure 1:**
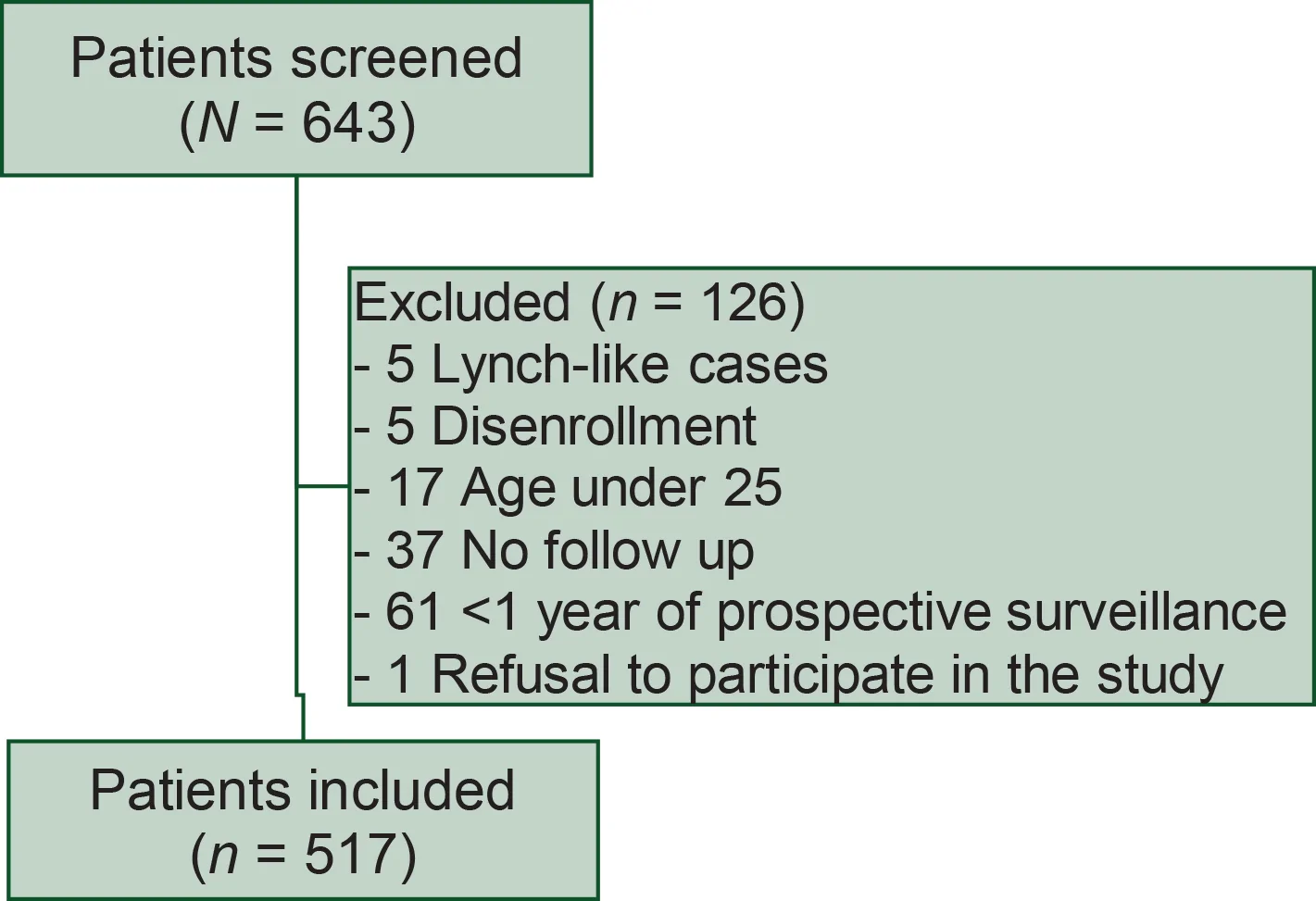
**Flow diagram illustration patient selection and applied exclusion criteria (final cohort: *N* = 517)**.

A total of 643 patients were initially screened for inclusion. After removal of duplicates and application of eligibility criteria, 517 patients carrying a P/LP variant in an MMR gene and enrolled in prospective follow-up were included in the final analysis. The patient selection process is summarized in Figure 1. Table 1 provides an overview of patient characteristics.

**Table 1:** Characteristics of the study population (*N =* 517)

Characteristics	*n* (%)
Median age at inclusion, years (IQR Q1; Q3)	43 (32; 52)
Sex	
Male	273 (52.8)
Female	244 (47.2)
No cancer at initiation of follow-up	281 (54.0)
Gene (P/LP)	
*MSH2*	191 (36.9)
*MLH1*	181 (35.0)
*MSH6*	90 (17.4)
*PMS2*	42 (8.1)
*EPCAM-MSH2*	12 (2.3)
*MLH1*, *EPCAM*	1 (0.2)

IQR, interquartile range; P/LP, pathogenic or likely pathogenic.

Regarding genetic distribution, 203 (39.3%) patients carried a P/LP variant in *MSH2* or *EPCAM*, 181 (35%) in *MLH1*, 90 (17.4%) in *MSH6*, and 42 (8.1%) in *PMS2*. One patient carried two distinct variants affecting both *EPCAM* and *MLH1*. The median age at inclusion was 43 years (IQR 32-52), and the age at last follow-up was 52 years (IQR 40-64). The cohort included 52.8% men and 47.2% women, with a median follow-up duration of 8 years (IQR 4-13). At the start of surveillance, 281 patients were cancer-free. A total of 201 cancers were diagnosed during prospective follow-up. Table 2 summarizes these cancers, with colon and rectum being the most frequent sites (*n =* 84; 41.8%).

**Table 2:** Types of cancer during follow-up (*N =* 201)

Cancer type	Number of cancers	Median age (Q1-Q3), years
Colon	65	55 (47-60)
Rectum	19	62 (52-68)
Cutaneous epidermoid	18	65 (52-69)
Ureter	16	59 (52-69)
Breast	12	59 (47-62)
Duodenum	7	55 (50-60)
Bladder	7	66 (62-71)
Pancreas	7	65 (62-72)
Prostate	6	59 (57-65)
Kidney	4	58 (49-67)
Sebaceous carcinoma	4	59 (59-61)
Endometrium	4	51 (49-53)
Jejunum	4	70 (64-74)
Glioblastoma	3	69 (67-72)
Basal cell	3	61 (58-66)
Gastric	3	71 (65-74)
Ovary	3	54 (47-65)
Lung	3	70 (68-73)
Biliary tract	2	63 (60-67)
Esophagus	2	63 (60-66)
Liver	2	60 (45-75)
Thyroid	1	56 (56-56)
Base of tongue	1	75 (75-75)
Chronic myeloid leukemia	1	66 (66-66)
Melanoma	1	77 (77-77)
Lymphoma	1	72 (72-72)
Leukemia	1	60 (60-60)
Anal canal	1	78 (78-78)

Most cancers (*n =* 183, 91%) occurred in patients aged >45 years (Supplementary Figure S1, available at https://doi.org/10.1016/j.esmogo.2026.100399). This observation applies only to incident cancers diagnosed after enrollment. Among the 125 patients who developed cancer, 50 (40%) had no prior cancer history, whereas 75 (60%) had a documented history of cancer before follow-up. Most patients developed only one cancer during follow-up (Supplementary Figure S2, available at https://doi.org/10.1016/j.esmogo.2026.100399). Cancers diagnosed before the start of prospective follow-up were excluded from the main analyses (Supplementary Table S1, available at https://doi.org/10.1016/j.esmogo.2026.100399). Of the 201 cancers diagnosed, 132 occurred in organs for which surveillance was recommended based on genetic predisposition. These included tumors of the upper GI and small bowel (stomach, duodenum, and jejunum; *n =* 14, 6.96%), lower GI tract (colon and rectum; *n =* 84, 41.8%), urinary tract (renal, bladder, and urothelial tumors; *n =* 27, 13.4%), and gynecologic cancers (*n =* 7, 3.48%). The cumulative follow-up amounted to 4827 person-years, yielding an overall annual incidence rate of 4.16% (95% CI 3.59-4.74).

**Figure 2:**
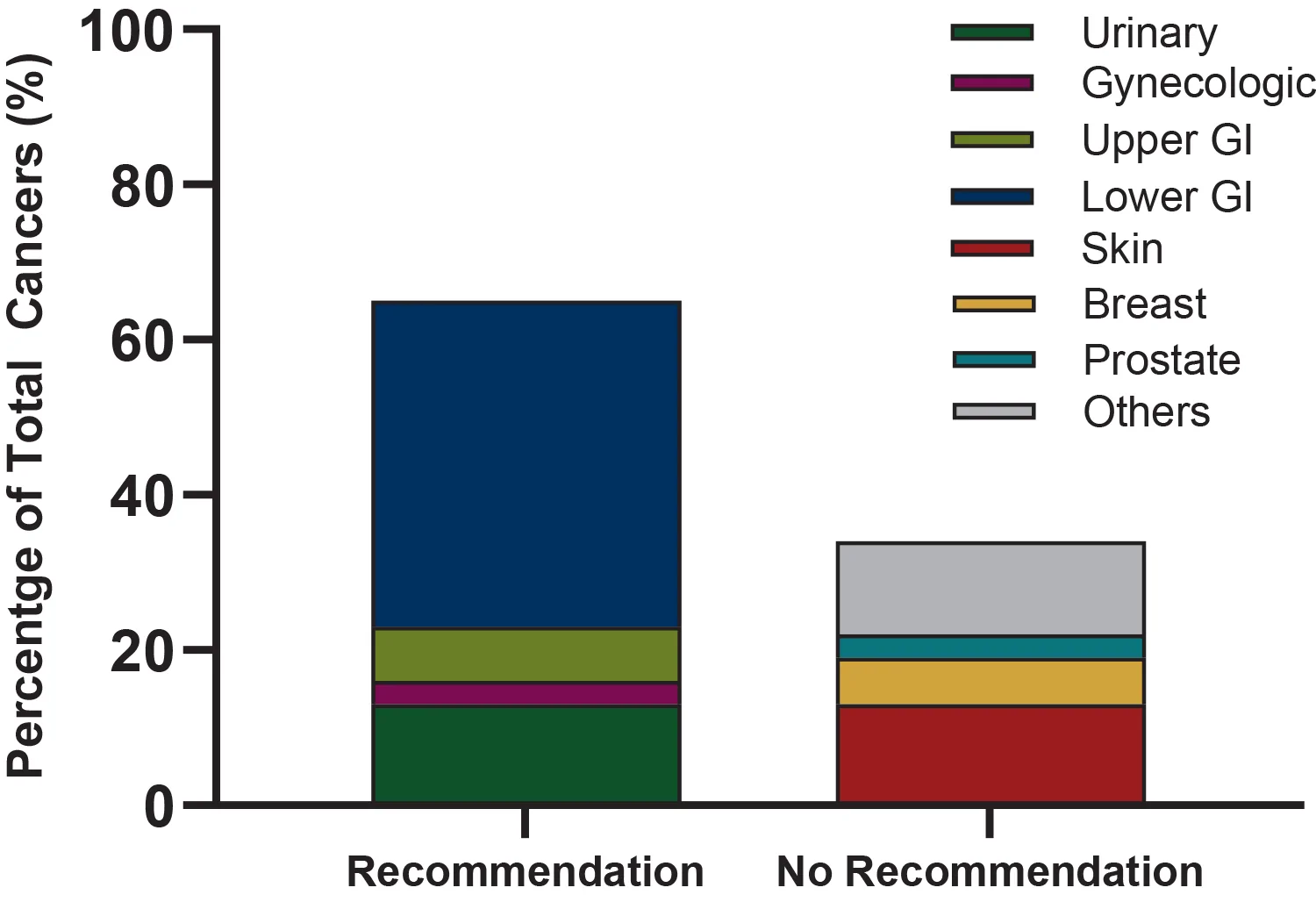
**Proportion of cancers inside versus outside guidelines-based surveillance (*N* = 201).**GI, gastrointestinal.

Among cancers in organs under guideline-based surveillance (*n =* 132), the overall incidence rate was 2.74% (95% CI 2.27-3.20), with organ-specific incidence rates as follows: lower GI tract, 1.74% (95% CI 1.37-2.11); urinary tract, 0.56% (95% CI 0.35-0.77); upper and median GI tract, 0.29% (95% CI 0.14-0.44); and gynecologic cancers, 0.15% (95% CI 0.04-0.25). Outside of the recommended surveillance organs, 69 cancers were diagnosed (1.43%, 95% CI 1.09-1.77). The most frequent sites were skin-related tumors (cutaneous, sebaceous, basal cell, or melanoma, *n =* 26; 0.54%, 95% CI 0.33-0.75), followed by breast (*n =* 12; 0.25%, 95% CI 0.11-0.39), pancreas (*n =* 7; 0.15%, 95% CI 0.04-0.25), and prostate (*n =* 6; 0.12%, 95% CI 0.03-0.22). Figure 2 shows the distribution of all 201 cancers according to whether a site-specific surveillance recommendation was applicable. Cancer-free survival from inclusion by gene is presented in Supplementary Figure S3, available at https://doi.org/10.1016/j.esmogo.2026.100399.

### Early detection of urinary, GI, and gynecologic tumors through routine surveillance in patients with Lynch syndrome

**Figure 3:**
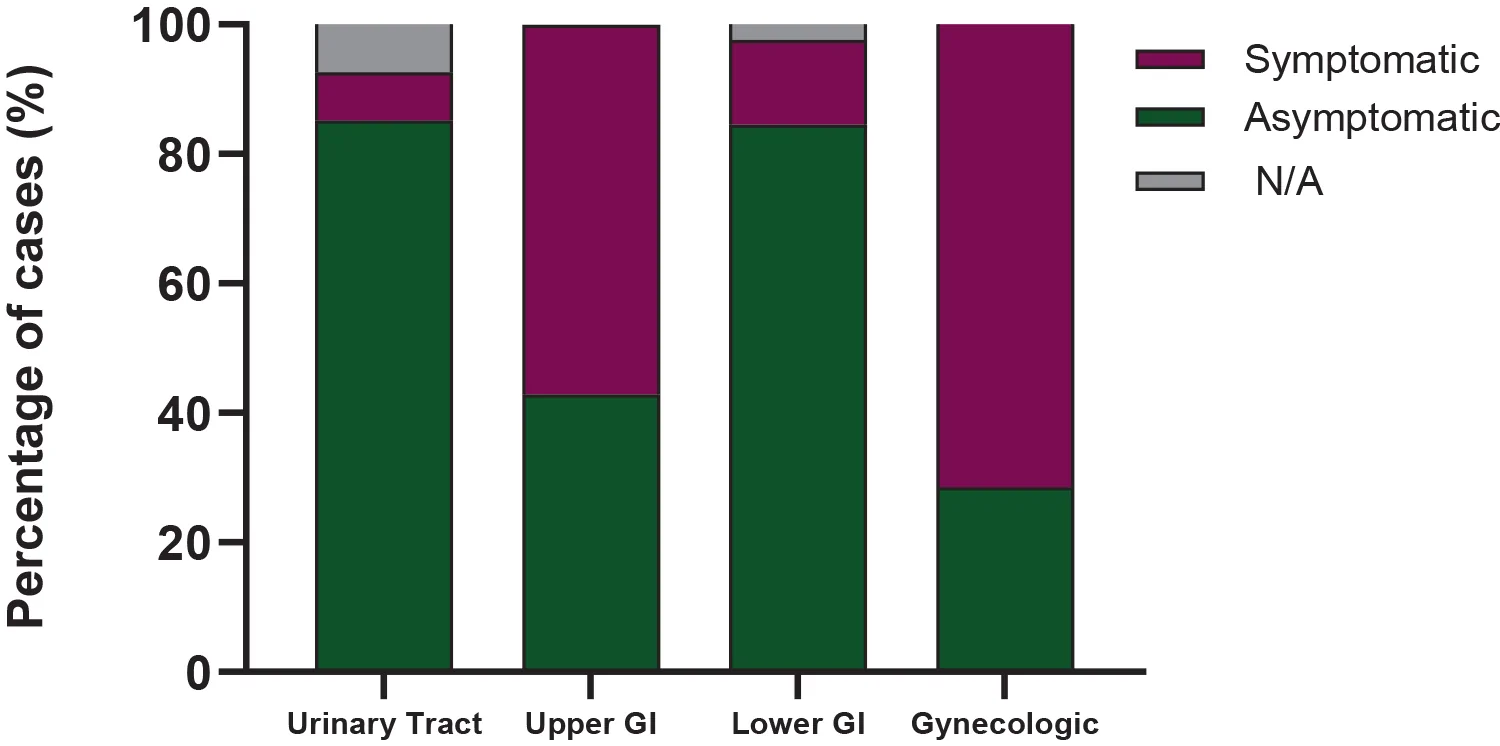
**Distribution of symptomatic and asymptomatic cancers diagnosed under recommendation during follow-up, by anatomical sphere.**GI, gastrointestinal; N/A, not applicable.

Among lower GI tumors (*n =* 71), the vast majority were diagnosed during scheduled surveillance colonoscopies, with only 11 cases detected due to symptoms. For upper GI tumors (*n =* 14), six were identified during routine endoscopic surveillance, whereas the remaining eight presented symptomatically. Urinary tract tumors were predominantly diagnosed asymptomatically (*n =* 23) through surveillance imaging, cytology, or incidental findings, with two cases diagnosed due to symptoms. Gynecologic cancers (*n =* 7) included two asymptomatic and five symptomatic cases. Overall, lower GI and urinary tract cancers occurring in organs with established surveillance recommendations were detected at asymptomatic stages, whereas this was less consistent for upper GI and gynecologic cancers, despite recommended surveillance (Figure 3).

Among the seven cancer-specific deaths in the recommendation group, five occurred in patients who were symptomatic at diagnosis (5.3%). In the no-recommendation group, nine cancer-related deaths were reported among 69 tumors (13.2%). Although this difference did not reach statistical significance (*P* = 0.059, two-tailed Fisher’s exact test), the odds of cancer-related death were lower in the recommendation group (odds ratio 0.37, 95% CI 0.13-1.03), suggesting a potential protective effect of surveillance (Supplementary Figure S4, available at https://doi.org/10.1016/j.esmogo.2026.100399).

### Upper and median GI cancers

During follow-up, 14 patients developed upper GI tumors. The most common tumor location was the duodenum (*n* = 7), followed by the jejunum (*n* = 4) and the stomach (*n* = 3). The median age at diagnosis was 60 years (IQR 52-64). Cancers were diagnosed during scheduled surveillance in six patients, whereas the remaining eight cases were identified symptomatically.

The median interval between the last upper GI endoscopy and cancer diagnosis was 12 months (IQR 8-15). Precancerous lesions, including adenomas or chronic gastritis, were detected during surveillance endoscopies in five patients before cancer diagnosis. Notably, three patients had a history of duodenal ulcers before developing duodenal cancers (*Helicobacter pylori*-positive: *n =* 1, *H. pylori-*negative, *n =* 2). Genetic analysis revealed that 10 cancers were associated with mutations in *MSH2* or *EPCAM*, three with *MLH1*, and one with *MSH6*; no tumors were linked to mutations in *PMS2*.

### Lower GI cancers

Among the 84 colorectal cancers (CRCs) diagnosed in 64 patients, 71 (84.5%) were detected during scheduled surveillance colonoscopies, whereas 11 (13.1%) were identified after the onset of symptoms. Two cases lacked documentation regarding the mode of diagnosis. Of the 64 patients, 46 presented with one cancer, 16 developed two cancers, and 2 developed three cancers during follow-up. The median age at diagnosis was 55 years (IQR 50-64). The median interval since the previous colonoscopy was 24 months (IQR 12-26). Lymph node involvement was observed in 19 cancers (16 in asymptomatic patients, three in symptomatic ones), with a median interval of 22 months since the previous colonoscopy, comparable to the interval observed in the overall cohort. The cumulative incidence of lower GI cancers during follow-up was similar between patients with or without a prior history of CRC (Supplementary Figure S5, available at https://doi.org/10.1016/j.esmogo.2026.100399). Regarding genetic distribution, among the 64 patients, 43 carried P/LP variants in *MSH2* or *EPCAM*, 18 in *MLH1*, and three in *MSH6*. No cases were associated with P/LP variants in *PMS2*.

### Urinary tract cancers

A total of 27 urinary tract tumors were diagnosed in 23 patients, with a median age at diagnosis of 61 years (IQR 54-70). Among these, 23 tumors were diagnosed in an asymptomatic setting, including three synchronous cancers in one patient and two metachronous cancers in patients with a prior history of urinary tract cancer. Two cancers were diagnosed symptomatically, and two cases lacked documentation regarding the mode of diagnosis. In asymptomatic cases, the primary triggers were urinary abnormalities [*n =* 11; hematuria (*n =* 8), abnormal cytology (*n =* 6), and both (*n =* 2)], imaging findings [*n =* 15; ultrasound (*n =* 10) and computed tomography/magnetic resonance imaging during surveillance for previous malignancies (*n =* 5)], both imaging and urinary abnormalities (*n =* 5), and cystoscopy (patients with prior urinary tract cancers, *n =* 2). At diagnosis, 22 of 23 asymptomatic tumors were localized, whereas 1 of the 2 symptomatic tumors was metastatic. Genetic analysis revealed P/LP variants in *MSH2* in 15 patients, *MLH1* in five, and *MSH6* in three, and no case was associated with P/LP variants in *PMS2*.

### Gynecologic data

Among the 244 women enrolled in the follow-up program, 53 had undergone prior gynecologic surgery before inclusion, including procedures carried out for malignant or benign conditions (Supplementary Data, available at https://doi.org/10.1016/j.esmogo.2026.100399). The remaining 191 women had not undergone gynecologic surgery at baseline and were included in the present analysis. These 191 women contributed a total of 1296 woman-years of follow-up. Follow-up was censored at the time of diagnosis of endometrial or ovarian cancer or at the time of prophylactic hysterectomy. The median duration of follow-up before censoring was 5 years (IQR 2-10). During follow-up, 86 of 191 women (45%) underwent prophylactic gynecologic surgery. The median age at surgery was 49.5 years (Q1: 46, Q3: 55). Overall, seven gynecologic cancers were diagnosed in six women during follow-up. This included four endometrial cancers and three ovarian cancers. One patient presented with synchronous endometrial and ovarian cancer. Five cancers were diagnosed in a symptomatic context (abnormal uterine bleeding or pelvic pain). One endometrial cancer was incidentally diagnosed on a prophylactic surgical specimen, and one ovarian cancer was detected in an asymptomatic patient based on an abnormality identified during routine pelvic ultrasound. The ages at diagnosis were 49, 49, 53, and 54 years for endometrial cancers, and 45, 54, and 69 years for ovarian cancers. All patients underwent surgery with curative intent, and no metastatic disease was observed at diagnosis. Pathogenic variants included mutations in *MSH2* (*n* = 3), *MLH1* (*n* = 2), and *MSH6* (*n* = 1), with no cases associated with *PMS2*.

## Discussion

This study provides a comprehensive overview of cancer incidence in patients with Lynch syndrome enrolled in a dedicated, structured surveillance program. Of the 643 patients initially screened, 517 carriers of a P/LP MMR variant were included in this retrospective analysis of prospectively collected data, with a median age of 43 years at inclusion and a median follow-up of 8 years. The genes most commonly affected were *MSH2/EPCAM* (39.3%) and *MLH1* (35%). Our findings underscore both the effectiveness and limitations of current surveillance strategies across multiple organ systems and highlight important implications for clinical management.

The prospective follow-up protocol and the comprehensive organ-specific surveillance strategies strengthen internal validity, minimizing recall bias and enhancing temporal accuracy. The cohort’s multiorgan perspective is particularly valuable, although some limitations inherent to retrospective analysis remain, including the inability to account for environmental or lifestyle risk factors and incomplete documentation of diagnostic details in isolated cases. External validity is further supported by the observed annual cancer incidence (4.16%), which aligns with international cohorts,^5^ and by the distribution of MMR variants, consistent with PLSD data (predominance of carriers of *MLH1* and *MSH2* variants).^6^

Despite high-quality colonoscopic surveillance, CRC remained the most frequent tumor type diagnosed during prospective follow-up in our cohort. Although colonoscopy is associated with reduced CRC-related mortality^7^ and improved adenoma detection over time,^8^ a nonnegligible proportion of cancers still arise under surveillance. Twenty percent of detected CRCs were node-positive (stage III), and cumulative incidence was similar in individuals with or without a prior CRC, in line with recent PLSD reports.^9^ These findings indicate that CRC continues to represent ∼40% of all cancers diagnosed in Lynch syndrome, with comparable cumulative incidences of first and metachronous CRC regardless of previous segmental resections.

Importantly, a 2-year colonoscopy interval and computer-aided detection systems, despite their value in average-risk populations, did not substantially improve early detection.^10^^,^^11^ Complementary approaches, including fecal microsatellite instability (MSI) testing, lower-threshold fecal immunochemical testing,^12^ and gut microbiome profiling^13^ may enhance early detection. These findings underscore that colonoscopy remains essential but is insufficient on its own.

Upper GI cancers were often diagnosed symptomatically (57%) despite surveillance, particularly in patients older than 50 years carrying pathogenic variants in *MLH1* or *MSH2*, an observation in line with recent reports.^14^ Increasing upper GI endoscopic surveillance frequency may be of limited benefit given the short interval between procedures (<1 year between the last endoscopy and cancer diagnosis), highlighting the need for complementary modalities, such as video capsule endoscopy or cross-sectional small bowel imaging.^15^ Reporting cases of duodenal ulcers preceding duodenal cancers is important, as such lesions currently do not trigger routine endoscopic reevaluation. Although anatomically, the jejunum is located beyond the ligament of Treitz and is classified as a part of the lower GI tract, it was grouped with upper GI tumors in this analysis due to its embryological and clinical proximity to lesions.

Urinary tract cancers, the second most common cancer, were largely asymptomatic (11.9%). Urinary tract surveillance in Lynch syndrome remains controversial.^16^ Our results suggest that low-cost, noninvasive testing from age 40 is appropriate, particularly for *MSH2* carriers, whereas dedicated surveillance for *PMS2* carriers appears unnecessary. Recently, urine MSI analysis demonstrated high sensitivity and specificity, detecting urothelial carcinoma in four patients, with no cancers detected over 12 months in those with a normal test.^17^ In contrast, conventional urine microscopy lacks adequate accuracy.^18^^,^^19^ Until urine MSI testing is routinely implemented, current urinary tract surveillance for patients with Lynch syndrome, particularly those with *MSH2* variants, should be maintained.

Gynecologic cancers were relatively rare in this cohort, likely reflecting both the widespread use of prophylactic hysterectomy and oophorectomy among high-risk women and the substantial number of endometrial and ovarian cancers diagnosed before inclusion. One incidental endometrial cancer was found during prophylactic surgery. Consistent with recent evidence, including a large Dutch cohort study of 1046 patients with Lynch syndrome,^20^ systematic gynecologic surveillance has not demonstrated clear benefit: stage at diagnosis and overall survival were similar between female patients who underwent surveillance and those who did not. These findings reinforce that timely prophylactic surgery effectively prevents endometrial and ovarian cancers, whereas routine surveillance for these organs remains of uncertain clinical value.

Among cancers not covered by current French Lynch syndrome guidelines, prostate, breast, and skin cancers were the most frequent, accounting for 44 of 69 cases (64%). Although prostate and skin cancers are recognized as part of the Lynch syndrome spectrum,^21^^,^^22^ they are not yet integrated in dedicated surveillance protocols. Population-based prostate-specific antigen screening from age 50 may be insufficient, as ∼10% of prostate cancers occur earlier,^23^ and more aggressive forms have been reported in Lynch syndrome.^24^ Given their frequency in the cohort, these cancers should be considered for inclusion in tailored Lynch syndrome prevention programs.

This study highlights three key messages with direct implications for Lynch syndrome. Firstly, the high annual cancer incidence (4.16%) and diverse tumor spectrum support follow-up within dedicated coordination centers, as fragmented, organ-specific care cannot adequately manage colorectal, urinary, upper GI, cutaneous, breast, and prostate malignancies. Multidisciplinary coordination is essential to ensure adherence, harmonize investigations, and interpret findings in the context of inherited risk. Secondly, surveillance does not fully prevent cancer, even in organs covered by established recommendations. CRC remained the most frequent malignancy, and more than half of upper GI cancers were diagnosed symptomatically. Gynecologic cancers were rare, likely reflecting the widespread use of prophylactic surgery rather than surveillance effectiveness. These findings underscore that surveillance enables primarily early detection rather than cancer elimination, with organ-specific effectiveness. Thirdly, a substantial proportion of cancers (34%) occurred outside guideline-recommended organs, notably skin, breast, and prostate. Ignoring these sites would miss an important proportion of clinically relevant cancers. With longer follow-up and increasing exposure to immune checkpoint inhibitors, future analyses may clarify how cancer latency and incidence are modified in this population.

### Conclusion

This study provides prospective evidence supporting the value of structured, multidisciplinary surveillance in patients with Lynch syndrome. The high cancer incidence and wide tumor spectrum observed over nearly 5000 person-years confirm that Lynch syndrome is a lifelong, multiorgan disease requiring coordinated expertise beyond isolated organ-based surveillance. Despite regular follow-up, cancers continue to occur across most organ systems, particularly in the colorectal and upper GI tract, highlighting the intrinsic limits of current surveillance. In contrast, the very low incidence of gynecologic cancers likely reflects the effectiveness of prophylactic surgery rather than surveillance itself, reinforcing its role as a cornerstone of prevention in high-risk women. Notably, more than one-third of cancers developed outside current surveillance recommendations, emphasizing that a substantial portion of the cancer burden remains inadequately addressed. These findings support the selective expansion of surveillance strategies to include organs such as the skin, prostate, and breast in their comprehensive care plans. Future prospective research should evaluate the role of innovative tools such as noninvasive liquid biopsies, which are being explored in the Validated non-invasive liquid biopsy tests for cancer PREDIction in LYNCH Syndrom (PREDI-LYNCH) program, to further enhance early detection and preventive strategies.
