# Supplementary material for: Structured surveillance in Lynch syndrome: effectiveness, limitations, and unmet needs
Source: ESMO Gastrointest Oncol. 2026 Aug 10;13(Pt B):100399. doi: 10.1016/j.esmogo.2026.100399 (PMC13519071; doi:10.1016/j.esmogo.2026.100399)
Supplement: Supplementary file 1 [file mmc1.docx]

**Supplementary Material**

**Supplemental Figure S1.** Distribution of Cancers by Age Group During Follow-up

**Supplemental Figure S2.** Distribution of Cancer Count per Patient during follow-up

Cancers per patients (Follow-up only)

**Supplemental Figure Bis:** Distribution of Cancer Count per Patient (Total Cohort)

Cancers per patients (Total Cohort)

**Supplemental Figure S3.** Cancer-free survival from inclusion.

The figure presents Kaplan-Meier survival curves estimating the probability of remaining free from a new cancer since inclusion in the follow-up program, stratified by gene. The number of patients at risk is shown below the x-axis for each 10-year interval, with breakdown by gene group. *EPCAM* carriers have been grouped with *MSH2* mutation carriers under the label “*MSH2*”

**Supplemental Figure S4.** Cancer-specific mortality by follow up recommendation group.

Cancer-Specific Mortality by Follow-up Recommendation Group

**Supplemental Figure S5.** Incidence of colorectal cancer over time.

Incidence rate of CRC during Follow-up

**Supplemental Table S1.** Type of cancer before follow-up (*n* =335).

| **Cancer Type** | **Number of Cancers** | **Median Age (IQR)** |
| --- | --- | --- |
| **Colon** | **207** | 45 (37–54) |
| **Endometrium** | **41** | 57 (51–59) |
| **Rectum** | **35** | 44 (34–55) |
| **Breast** | **12** | 55 (35–80) |
| **Ovary** | **6** | 42 (38–45) |
| **Gastric** | **4** | 54 (52–57) |
| **Sebaceous carcinoma** | **4** | 56 (52–62) |
| **Prostate** | **4** | 64 (63–68) |
| **Basal cell** | **3** | 58 (54–60) |
| **Ureter** | **2** | 72 (68–74) |
| **Jejunum** | **3** | 45 (43–55) |
| **Cutaneous epidermoid** | **3** | 54 (45–64) |
| **Melanoma** | **2** | 26 (18–34) |
| **Gallbladder** | **1** | 57 (57–57) |
| **Pancreas** | **1** | 53 (53–53) |
| **Uterine** | **1** | 28 (28–28) |
| **Renal pelvis** | **1** | 52 (52–52) |
| **Thyroid** | **1** | 44 (44–44) |
| **Duodenum** | **1** | 48 (48–48) |
| **Testis lymphoma** | **1** | 68 (68–68) |
| **Kidney** | **1** | 68 (68–68) |
| **Ampulla of Vater** | **1** | 44 (44–44) |
